# Phylogenetic taxonomy of the Zambian Anopheles coustani group using a mitogenomics approach

**DOI:** 10.21203/rs.3.rs-5976492/v1

**Published:** 2025-04-07

**Authors:** Soha Usmani, Mary E. Gebhardt, Limonty Simubali, Kochelani Saili, Westone Hamwata, Hunter Chilusu, Mbanga Muleba, Conor J. McMeniman, Anne C. Martin, William J. Moss, Douglas E. Norris, Reneé L.M.N. Ali

**Affiliations:** The W. Harry Feinstone Department of Molecular Microbiology and Immunology, Johns Hopkins Bloomberg School of Public Health; The W. Harry Feinstone Department of Molecular Microbiology and Immunology, Johns Hopkins Bloomberg School of Public Health; Macha Research Trust; Macha Research Trust; Tropical Diseases Research Centre; Tropical Diseases Research Centre; Tropical Diseases Research Centre; The W. Harry Feinstone Department of Molecular Microbiology and Immunology, Johns Hopkins Bloomberg School of Public Health; Department of Epidemiology, Johns Hopkins Bloomberg School of Public Health,; Department of Epidemiology, Johns Hopkins Bloomberg School of Public Health,; The W. Harry Feinstone Department of Molecular Microbiology and Immunology, Johns Hopkins Bloomberg School of Public Health; The W. Harry Feinstone Department of Molecular Microbiology and Immunology, Johns Hopkins Bloomberg School of Public Health

**Keywords:** Anopheles coustani, mitochondrial genome, phylogeny, Zambia, malaria

## Abstract

**Background:**

Mosquito species belonging to the *Anopheles coustani* group have been implicated in driving residual malaria transmission in sub-Saharan Africa and are regarded as an established primary vector in Madagascar. The morphological identification of mosquitoes in this group is challenging due to cryptic features and their molecular confirmation is difficult due to a paucity of reference sequence data representing all members of the group. Conventional molecular barcoding with the cytochrome oxidase I (COI) gene and the internal transcribed spacer 2 (ITS2) region targets is limited in their discrimination and conclusive identification of members of species complexes. In contrast, complete mitochondrial genomes (mitogenomes) have demonstrated much improved power over barcodes to be useful in rectifying taxonomic discrepancies in Culicidae.

**Methods:**

We utilized a genome skimming approach via shallow shotgun sequencing on individual mosquito specimens to generate sequence reads for mitogenome assembly. Bayesian inferred phylogenies and molecular dating estimations were perfomed on the concatenated protein coding genes using the Bayesian Evolutionary Analysis by Sampling Trees 2 (BEAST 2) platform. Divergence estimates were calibrated on published calucations for *Anopheles-Aedes*.

**Results:**

This study generated 17 new complete mitogenomes which were comprable to reference *An. coustani* mitogenomes in the GenBank repository by having 13 protein coding, 22 transfer RNA and 2 ribosomal RNA genes, with an average length of 15,400 bp and AT content of 78.3%. Bayesian inference using the concatenated protein coding genes from the generated and publicly available mitogenomes yielded six clades: one for each of the four taxa targeted in this study, the GenBank references, and a currently unknown species. Divergence times estimated that the *An. coustani* group separated from the *An. gambiae* complex approximately 110 million years ago (MYA), and members within the complex diverged at times points ranging from~34 MYA to as recent as ~7 MYA.

**Conclusions:**

These findings demonstrate the value of mitochondrial genomes in differentiating cryptic taxa and help to confirm morphological identities of *An. coustani s.s., An. paludis, An. zeimanni* and *An. tenebrosus*. Divergence estimates with the *An. coustani* group are similar to those for well-studied anopheline vector groups. These analyses also highlight the likely prescence of other cryptic *An. coustani* group members circulating in Zambia.

## Background

Vector control methods like indoor residual spraying (IRS) and long-lasting insecticidal nets (LLINs) have been instrumental in progress toward malaria elimination [1, 2]. Primary, well-studied vectors like *Anopheles gambiae* and *An. funestus*, which typically engage in endophagic and endophilic behaviors by seeking human hosts indoors, are the focus of these key intervention measures [1, 2]. However, selection pressure driven by the broad deployment of IRS and LLINs have either reduced these populations, driven insecticide resistance, yielded shifts in vector species composition and/or resulted in changes in biting and resting behaviors [2–7]. Shifts to outdoor biting or having a high plasticity in this behavior, and the existence of other exophagic malaria vectors have been identified as a significant barriers to malaria control and elimination [3, 8, 9]. Though frequently collected, exophagic anopheline mosquitoes such as members of the *An. coustani* group [10–12], *An. squamosus*, and *An. rufipes* [14] are understudied despite contributing to malaria transmission in sub-Sahran Africa.

The *Anopheles coustani* group is widely distributed throughout sub-Saharan Africa and the Middle East, with members typically exhibiting zoophilic and outdoor foraging behaviors [11]. Within the group, morphologically similar species including *An. coustani, An. zeimanni, An. paludis*, and *An. tenebrosus*, have demonstrated opportunistic foraging towards anthropophilic and endophilic feeding [10, 15]. Little is known about the basic biology, ecology and behaviors of most of these species. This knowledge gap is particularly noteworty given members of the group have been implicated as established vectors with a key role in sustaining residual malaria transmission in Kenya, Madagascar, Ethiopia, Cameroon, Mozambique and Zambia [10, 15–20]. Members of this group present an imminent threat to malaria elimination efforts due to inherent plasticity in their foraging behaviors, which enable them to evade many of the existing vector control strategies that target endophagic and endophilic mosquitoes [3, 21–23].

Morphological and molecular techniques have proved to be challenging for identification of species in this group due to cryptic features, damaged specimens which obscures key morphological attributes [23–25], and the paucity of reference molecular data for comparison in genomic repositories [26]. Additionally, the well-established cytochrome oxidase I gene (COI) and the internal transcribed spacer 2 (ITS2) molecular barcodes commonly used for species confirmation have limited power in delineating phylogenetic disparities in cryptic species groups [23, 27, 28]. Though limited in number, published genetic and molecular studies have highlighted cryptic members within the *An. coustani* group [29–31]. Early studies using chromosomal inversion analyses identified *An. coustani* and *An. crypticus* as separate species [29, 30]. Genetic diversity analyses in Zambia and the Democratic Republic of the Congo also reported two distinct phylogenetic groups of *An. coustani* populations [31] in 2020, and definitive species identification remained unverified based on conventional barcoding methods in Mozambique in 2024 for *An. tenebrosus* and *An. Zeimanni* [18].

Mitochondrial genomes (mitogenomes) are circular, double stranded DNA molecules with high copy numbers, low incidence of recombination, absence of introns, and maternal inheritance [32–34]. These characteristics facilitate utility for inferring phylogenies, addressing species identification, and evolutionary studies in a range of organisms including metazoans [35–37]. The mitogenome encodes for 13 protein coding genes (PCGs), 22 transfer RNA (tRNA), 2 ribosomal RNA (rRNA) and a non-coding control region [38]. Developments in computational and sequencing technologies enable more datasets to include chromosomal and mitochondrial reference genomes for mosquito species, where both data are available [32, 36, 39, 40]. However, sequencing efforts to date have been biased toward well-studied and defined species groups such as *An. gambiae* [41–43] and *An. funestus* [44, 45].

At the present time, there are five mitochondrial and two chromosomal genomes collectively available in the GenBank databse for *An. coustani* sensu stricto and *An. ziemanni*[46–49]. Generating additional reference mitogenomes for members of the *An. coustani* group would prove beneficial for phylogenetic analyses and these data can inform taxonomic classification, mosquito diversity, and evolutionary history in relation to malaria transmission of this understudied group [50, 51]. Although full genomes would be ideal for these tasks, mitochondrial genomes can be sequenced and assembled quickly and inexpensively compared to full nuclear genome sequencing and annotation.

Given that accurate species identification is crucial for vector incrimination and the development and evaluation of vector control strategies, the taxonomic resolution of species in the *An. coustani* group is essential for malaria control efforts [23]. Additionally, it is not plausible to generate significant inferences regarding population and evolutionary histories or actual taxonomic species boundaries based on currently available evidence. This study aims to contribute complete reference mitochondrial genomes for members of the *An. coustani* group in Zambia and delineate the phylogenetic taxonomy for this epidemiologically important mosquito complex.

## Methods

### Mosquito collection and morphological identification.

**Figure 1:**
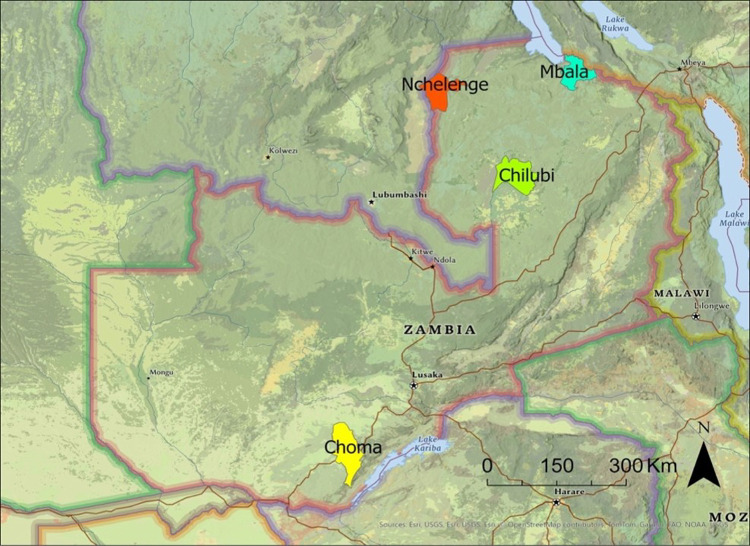
Map showing the four Districts used for mosquito collections in this study study.

Outdoor mosquito collections were carried out in Zambia as part of the Southern and Central Africa International Centers of Excellence for Malaria Research (ICEMR). Specimen collections were performed in 2023–2024 using standard Centers for Disease Control and Prevention (CDC) miniature light traps in Choma and Nchelenge Districts (Fig. 1). Larvae were collected in the Chilubi and Mbala Districts and were reared to adults at the Tropical Diseases Research Centre (TDRC), Ndola, Zambia. Mosquitoes were sorted and identified using a morphological key [52] by members of the ICEMR team. Specimens morphologically identified as *An. coustani, An. ziemanni, An. tenebrosus*, and *An. paludis* were stored in tubes containing silica gel and shipped to the Johns Hopkins Bloomberg School of Public Health (Maryland, USA) for molecular analysis. The specimens with intact morphological characteristics that allowed clear identification as *An. coustani, An. tenebrosus, An. paludis* and *An. ziemanni*, were molecularly confirmed and selected for sequencing and downstream analysis. Specimens that could not be further keyed to species type due to damage or cryptic features were labelled as *An. coustani* sensu lato (s.l.).

### DNA extraction, sequencing, mitogenome assembly and annotation.

Single mosquito specimens were homogenized in a mixture containing 98 μL of PK buffer (Applied Biosystems, Waltham, MA) and 2 μL of proteinase K (Applied Biosystems, Waltham, MA) followed by an incubation at 56°C for 2.5 hours [53]. After incubation, DNA was extracted using the Qiagen DNeasy Blood and Tissue kit (Qiagen, Hilden, Germany) as per the manufacturer’s instructions. Using the Qubit dsDNA assay kit (Thermo Fisher Scientific, Waltham, MA) the extracted DNA was quantified and stored at −20°C. Extracted DNA was shipped to SeqCenter (Pittsburgh, USA) for library construction and Illumina sequencing. Libraries were 150 bp paired end sequenced to a depth of 13.3 million reads.

Using NOVOPlasty [54] (RRID:SCR_017335) version 4.3.5, the mitochondrial genomes were assembled with k-mer set at 39 and reference mitogenomes (MT_806097, NC_064609, NC_064611) as seed sequences. The generated contigs were automatically annotated using the MITOchondrial genome annotation (MITOS) [55] galaxy tool under the invertebrate genetic code with default settings. Using reference *An. coustani* mitochondrial genomes as guides, start and stop codon positions were manually modified in Geneious Prime (RRID:SCR_010519) version 2023.2.1 (Biomatters, Auckland, Australia). Resulting sequences and their corresponding annotations were uploaded to the GenBank database.

#### Phylogenetic analysis and divergence time estimation

The protein coding genes of the mitogenomes constructed in this study and those from *An. coustani* (MT_806097, NC_064611, OX_030899), *An. ziemanni* (NC_064609, OX_030922), *An. gambiae* (NC_083487), *An. arabiensis* (NC_028212), *An. pharoensis* (PP_068257), *An. rufipes* (PP_068269) and *Ae. aegypti* (NC_035159) reference sequences were imported from the GenBank repository, aligned, and exported in nexus format using the MAFFT amino acid alignment mode in Geneious Prime (RRID:SCR_010519) version 2023.2.1 (Biomatters, Auckland, Australia). Using jModelTest (v2.1.10) software [56] with default settings. The best fit base pair substitution model for the aligned sequence matrix was identified based on the Bayesian information criterion (BIC) and the Akaike information criterion (AIC). Bayesian inference analysis and node age calculations were performed in Bayesian Evolutionary Analysis by Sampling Trees (BEAST) version 2.7.6 [57] using the GTR + G + I substitution model with three independent runs as described [58]. An application of 20% burn-in rate was implemented for tree building purposes and FigTree v.1.4.4 (http://tree.bio.ed.ac.uk/software/figtree/) was used to visualize trees. Molecular dating time estimations were inferred alongside the previously mentioned parameters using *Aedes-Anopheles* divergence time as the calibration point. The *Aedes-Anopheles* divergence was set as a prior with normal distribution around 154.7 million years ago (MYA) [59]. Pairwise genetic distances between representative groups were computed in the MEGA X 10.0.5 software [60] using the exported MAFTT amino acid alignment from Geneious Prime.

## Results

### Mitochondrial genome characteristics

**Figure 2:**
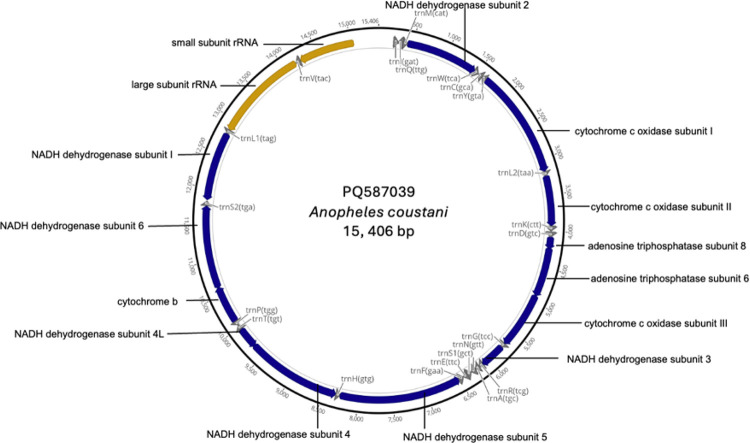
Representative mitochondrial genome of the *An. coustani* group comprising 37 genes: 13 PCGs, 22 tRNAs and 2 rRNAs.

Review of collections from 2023–2024 provided 81 putative *An. coustani* group specimens. From these, 17 specimens passed morphological and molecular confirmation, and were sequenced and annotated. The 17 novel mitogenomes produced in this study were arranged similarly to the reference *An. coustani* and *An. ziemanni* mitochondrial genomes available in the GenBank database, with lengths ranging from 15,404 bp (An. *tenebrosus*) to 15,425 bp *(An. paludis)* and an average AT content of 78.3% (Table S1). The *An. coustani* group mitogenomes comprised of 13 PCGs, 22 transfer RNAs (tRNAs) and 2 ribosomal RNAs (rRNAs) as shown in Fig. 2.

### Phylogenetic and Divergence time analysis

**Figure 3:**
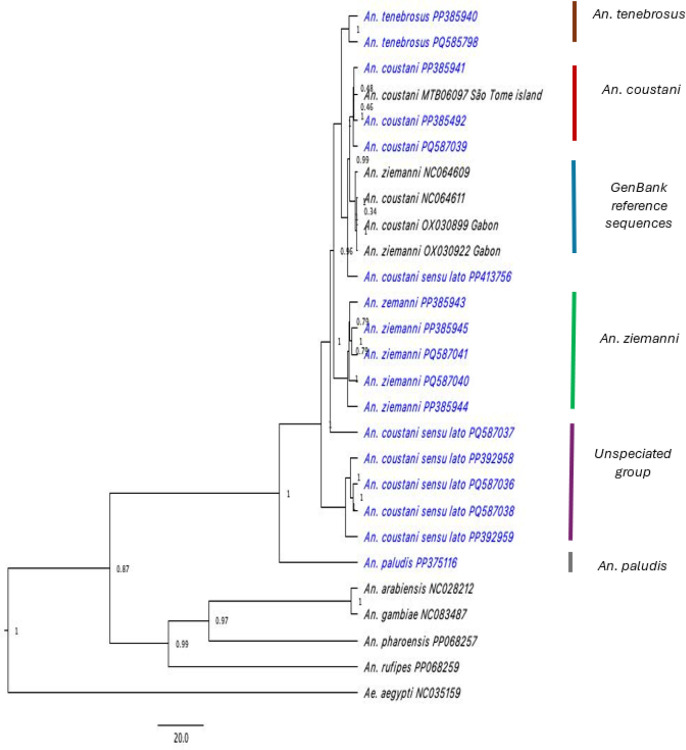
Bayesian tree showing phylogenetic relationships of 17 new mitogenomes (highlighted in blue) of the *An. coustani* group with other *Anopheles* species. The tree was constructed using the concatenated PCGs using BEAST v 2.7.6 as described in the methods. The posterior probabilities supporting the tree topology are represented by the values at the nodes

The aligned and concatenated protein coding sequences from the 25 mitogenomes (24 *Anopheles* and 1 *Aedes* mosquito species as an outgroup) resulted in a matrix of 11,023 bp, which was included in the Bayesian analyses for the phylogenetic tree construction and molecular dating. Bayesian inferences resulted in well supported phylogenies with posterior probabilities close to or at one for the mitogenomes generated in this study. Six main clades were identified. Five clades represent four taxa *(An. tenebrosus, An. coustani, An. ziemanni, An. paludis*) from *the An. coustani* group and an ‘unspeciated’ group comprised of specimens morphologically identified as *An. coustani* s.l. (Fig. 3). The sixth clade is comprised of the GenBank reference sequences labeled as *An. coustani* and *An. ziemanni* as identified in GenBank.

**Figure 4:**
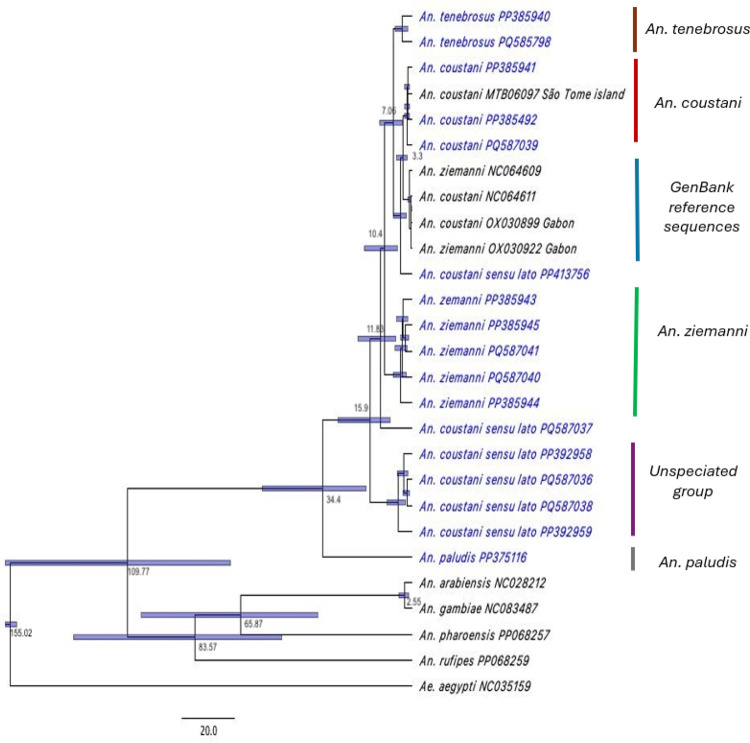
Phylogenetic tree showing inferred molecular divergence estimates (MYA) for members of the *An. coustani* group (highlighted in blue) using the concatenated PCGs from mitogenomes generated in this study. The mean divergence time (MYA) predicted for each event is represented by the values at the tree nodes. The bars show the 95% confidence intervals.

The most recent common ancestor (MRCA) of all *Anopheles* was dated at 109.77 MYA (Fig. 4) with a 95% confidence interval spanning from 68.4 to 157.02 MYA (Table 1), using the *Anopheles-Aedes divergence* period set at 154.7 MYA [59]. The MRCA for *An. coustani* s.l. and *An. ziemanni* within the *An. coustani* group dates to 10.4 MYA, with a credibility interval that spans from 0.7 to 14.3 MYA. This MRCA is more recent than those determined for the unspeciated group and *An. paludis* from other members of the *An. coustani* group, estimated at 15.9 and 34.4 MYA respectively (Fig. 4 and Table 1). The pairwise genetic distance matrix calculations (Table S2) between representatives of each group/clade ranged from 0.0008–0.0217, except for *An. paludis* which resulted in a much wider genetic distance.

## Discussion

This study generated 17 new full-length mitochondrial genomes for members of the *An. coustani* group from Zambia that improve the resolution of within-group species taxonomy and provide insight into the species group’s complexity. Bayesian analyses using the concatenated PCGs from the mitogenomes generated in this study supported phylogenies and separated the specimens into distinct taxonomic groups including *An. coustani* s.s., *An. tenebrosus, An. paludis* and *An. ziemanni*. These new phylogenies have better taxonomic resolution and stronger branch support when compared to earlier studies in Zambia using the COI and ITS2 molecular barcodes [31, 61]. Those studies separated *An. coustani* s.l. specimens into two general groups, *An. coustani* clade 1 or 2 [31, 61], or undefined *Anopheles* species groups [61]. Furthermore, a subset of the *An. coustani* s.s. specimens in this study formed a separate clade from the GenBank reference genome sequences identified as *An. coustani* and *An. ziemanni*, an indication of additional complexity within the *An. coustani* species group or perhaps, morphological misidentification prior to sequencing.

This study highlights the significance of anopheline morphological data and molecular verification for identifying both known and unknown anopheline species, especially those implicated as malaria vectors. Though previous studies have shed light on mosquitoes in the *An. coustani* group and their association with malaria transmission [20, 21, 23, 31], there remains a paucity of sequence data corresponding to well-curated specimens which can be used to accurately speciate members of this group. As a result, the majority of available COI and ITS2 sequences are categorized as *‘An. coustani* s.l.’, rather than to specific species within the group [18, 24, 61].

Despite the increased taxonomic power the data in this study provided, there were some limitations to identification of all specimens. In the absence of voucher specimens available for sequencing or genomic data for other members of the group such as *An. caliginosus, An. crypticus, An. namibiensis* and *An. symesi* [11] our study faced challenges in determining the phylogenetic placement and species identification for one clade of specimens, which we designated as *An. coustani* s.l. These mosquito specimens were collected primarily in Nchelenge District on the border with the Democratic Republic of the Congo (DRC) where *An. caliginosus* has been reported [11, 62], suggesting this species or perhaps other members of the *An. coustani* group may be more widely distributed in Zambia. Another caveat is the indistinguishable morphological features of adult female *An. crypticus* and *An. coustani* s.s. mosquitoes [11, 30]. It is possible that the *An. coustani* s.s. specimens sequenced in this study, or alternatively the GenBank references, represent *An. crypticus*. This was implied by a study that identified ‘An. *coustani* clade 2’ as putative *An. crypticus* [61]. Furthermore, pairwise distance estimates between representatives from these two groups suggest the potential prescence of *An. crypticus* circulating in Zambia. However, with the lack of reference specimens and the documented species range limited to South Africa [11, 30], it is problematic to verify the presence of this species or correlate molecular and cytogenetic data to morphological identifications across different species and studies.

Genetic distance matrices may provide definition of species boundaries [63], and the calculations derived from this study reinforce the complexity of relatedness among species such as *An. coustani* and *An. ziemanni*, further implying that cryptic speciation may be due to behavioral and ecological preferences [64]. Although studies for African anophelines have been biased towards well-recognized vectors such as *An. funestus* and *An. gambiae* [43, 45, 65], divergence estimations and phylogenies are also reported to be unresolved due to complexities such as introgression [25, 58, 66]. Our molecular divergence calculations suggest the *An. coustani* group diverged from the *An. gambiae* species complex ~110 MYA. This is consistent with inferences made by previous studies which reported the last common ancestor of *Anopheles* ~100 MYA [67] and the African distribution of the *Anopheles* subgenus ~113 MYA [68]. Molecular dating based on this phylogenetic analysis shows *An. paludis* splitting ~ 34 MYA from closely related species group members. This divergence time is older than that estimated between the other clades and like that for *An. gambiae* and *An. funestus*, suggests that reproductive or opportunistic behavioral adaptions may have occurred to explain why some species group members may be more involved in the transsion of *Plasmodium falciparum*.

## Conclusions

This is the first publication using a genome skimming strategy to generate 17 mitochondrial genomes for representatives of the *An. coustani* group. We were able to estimate divergence times for members of the group for which there is data and this study emphasizes the importance of actively pursuing accurately identified morphological voucher specimens for molecular characterization collected from other African regions. This is required for the clear delineation of species boundaries as well as for the taxonomic rectification among *An. coustani* members which have been shown to be closely related in this study. These findings also highlight the need for study of the basic biology of this group, inlcuding reproductive compatibility between members of the group which may resolve some of the taxonomic mysteries and most critically, their biological capacity to vector human pathogens is largely unknown. With changes in land use, climate and the decrease or shifts in primary malaria vector populations, research should focus on the ecological and behavioral characteristics of species in this and similarly understudied anopheline groups, as their importance in malaria transmission becomes more prominent.

## Figures and Tables

**Table 1:** Divergence estimations output from BEAST v 2.7.6 for *Anopheles* species including the mitochondrial genomes generated in this study.

Selected nodes	Mean ages	95% credibility interval
*Aedes/Anopheles*	155.02	150.00 −158.02
*Anopheles*	109.77	68.40–157.02
*An*. paludis/*An*. *coustani*group	34.40	17.40–57.00
Unspeciated group/*An*. *coustani* group	15.90	7.20 −24.10
*An. coustani s.s./An. ziemanni*	10.40	0.70 −14.30
*An. coustani* s.s./*An*. *tenebrosus*	7.06	3.00 −12.50

## Data Availability

The dataset supporting the conclusions of this article are available in the GenBank repository under the BioProject PRJNA1061161. The mitochondrial genomes are available with accession numbers PP375116, PP385940-PP385945, PP392958-PP392959, PP413756, PQ585798 and PQ587036-PQ587041.

## Abbreviations

IRS: Indoor residual spraying
LLINs: Long lasting insecticidal nets
COI: Cytochrome oxidase I
ITS2: Internal transcribed spacer 2
PCGs: Protein coding genes
tRNA: transfer RNA
rRNA: ribosomal RNA
ICEMR: International Centers of Excellence for Malaria Research
CDC: Centers for Disease Control and Prevention
MRT: Macha Research Trust
TDRC: Tropical Diseases Research Centre
BIC: Bayesian information criterion
AIC: Akaike information criterion
BEAST: Bayesian Evolutionary Analysis by Sampling Trees
MYA: Million years ago
MRCA: Most recent common ancestor
DRC: Democratic Republic of Congo
